# The added value of C-reactive protein to clinical signs and symptoms in patients with obstructive airway disease: results of a diagnostic study in primary care

**DOI:** 10.1186/1471-2296-7-28

**Published:** 2006-05-02

**Authors:** Antonius Schneider, Geert-Jan Dinant, Inko Maag, Lutz Gantner, Joachim Franz Meyer, Joachim Szecsenyi

**Affiliations:** 1 University Medical Hospital Heidelberg, Dept. of General Practice and Health Services Research; Heidelberg, Germany; 2 University Maastricht, Care and Public Health Research Institute, Dept. of General Practice, Maastricht, the Netherlands; 3 University Medical Hospital Heidelberg, Dept. of Cardiology, Angiology and Pneumology, Heidelberg, Germany

## Abstract

**Background:**

To evaluate the diagnostic accuracy of clinical signs and symptoms, C-reactive protein (CRP) and spirometric parameters and determine their interrelation in patients suspected to have an obstructive airway disease (OAD) in primary care.

**Methods:**

In a cross sectional diagnostic study, 60 adult patients coming to the general practitioner (GP) for the first-time with complaints suspicious for obstructive airway disease (OAD) underwent spirometry. Peak expiratory flow (PEF)-variability within two weeks was determined in patients with inconspicuous spirometry. Structured medical histories were documented and CRP was measured. The reference standard was the Tiffeneau ratio (FEV_1_/VC) in spirometry and the PEF-variability. OAD was diagnosed when FEV_1_/VC ≤ 70% or PEF-variability > 20%.

**Results:**

37 (62%) patients had OAD. The best cut-off value for CRP was found at 2 mg/l with a diagnostic odds ratio (OR) of 4.4 (95% CI 1.4–13.8). Self-reported wheezing was significantly related with OAD (OR 3.4; CI 1.1–10.3), whereas coughing was inversely related (OR 0.2; CI 0.1–0.7). The diagnostic OR of CRP increased when combined with dyspnea (OR 8.5; 95% CI 1.7–42.3) or smoking history (OR 8.4; 95% CI 1.5–48.9). CRP (p = 0.004), FEV_1 _(p = 0.001) and FIV_1 _(p = 0.023) were related with the severity of dyspnea. CRP increased with the number of cigarettes, expressed in pack years (p = 0.001).

**Conclusion:**

The diagnostic accuracy of clinical signs and symptoms was low. The diagnostic accuracy of CRP improved in combination with dyspnea and smoking history. Due to their coherence with the severity of dyspnea and number of cigarettes respectively, CRP and spirometry might allow risk stratification of patients with OAD in primary care. Further studies need to be done to confirm these findings.

## Background

The impact of obstructive airway diseases (OAD) is increasing, leading to high disability and mortality [1]. Consequently, optimal diagnostic performance is becoming an increasing challenge in primary care [2]. Medical history taking and physical examination deliver important information for diagnosing obstructive airway diseases (OAD) in primary care, but their predictive values are weak [3,4]. Attempts were made to improve the diagnostic accuracy in this field, like the single measurement of peak expiratory flow (PEF) and the use of screening questionnaires [5,6], but their sensitivities or specificities were low. Therefore, it would be helpful to find additional clinical markers to identify patients with risk for having an obstructive airway disease and to estimate their severity of the disease. One useful predictor for OAD could be C-reactive protein (CRP), as it is related with irreversible airway obstruction [7] due to some kind of systemic inflammation [8].

However, the core of diagnostics is the spirometry and many efforts are done to implement its use in primary care [9,10]. Consequently, spirometers are increasingly available in general practice, and modern instruments are not only measuring FEV_1 _(forced expiratory volume in one second) and VC (vital capacity), but also inspiratory parameters. Inspiratory parameters could be of value for the assessment of the disease, as FIV_1 _(forced inspiratory volume in one second) was shown to be related with the severity of dyspnea [11,12]. The aim of the present study was to determine the diagnostic accuracy of clinical signs and symptoms and CRP for diagnosing airway obstruction in primary care. A secondary aim was to identify predictors for the severity of the disease by analyzing the relation between clinical signs and symptoms, CRP and spirometric parameters.

## Methods

### Design

This cross sectional diagnostic study was performed between October 2003 and May 2004 in six general practices. The results from spirometry (FEV_1_/VC = Tiffeneau-Quotient) and the variability of PEF-measurement within two weeks were used as a reference standard.

### Participants

Sixty adult patients coming to the general practitioner for the first-time with complaints leading to the assumption of some kind of OAD were included consecutively. They presented symptoms like dyspnea, coughing, expectoration or self-reported wheezing. The patients have not been diagnosed earlier for OAD and they have not received any anti-obstructive medicine before. Other exclusion criteria related to well known side effects of inhaled sympathomimetics, namely untreated hyperthyreosis, unstable coronary artery disease or cardiac arrhythmia. Pregnancy also leads to exclusion. The study was approved by the Medical Ethics Committee of the University Heidelberg. Patients gave written informed consent.

### Measurements

Medical history was taken with a structured interview similar to the asthma screening questionnaire of the European Community Respiratory Health Survey (ECRHS) [13]. This questionnaire was designed to assess the prevalence of asthma (= reversible OAD) in several European areas. The interview was expanded to symptoms of COPD (= irreversible OAD) (table 1). Patients were asked for the severity of their dyspnea, categorized as dyspnea at rest (severe); dyspnea while walking straight ahead (moderate); dyspnea while taking the stairs of two floors (mild) and no dyspnea. Patients were questioned about their smoking habits, allowing calculation of the number of pack years (= [years of smoking: 20] × number of daily cigarettes). The physician performed the physical examination, always including auscultation of the patients' lungs. All findings were documented and checked for completeness.

**Table 1:** Characteristics of the study population Values are numbers (proportion) or mean (SD); OAD = obstructive airway disease

	**Overall**	**No OAD or restrictive pattern**	**Reversible OAD**	**Not reversible OAD**
	**n (%)**	**n (%)**	**n (%)**	**n (%)**
Total study population	60 (100)	23 (38)	27 (45)	10 (17)
Female	40 (67)	15 (65)	19 (70)	6 (60)
Age [mean (SD)]	46 (18)	42 (18)	46 (17)	54 (18)
Have you had wheezing in your chest (yes)	39 (65)	11 (48)	22 (82)	6 (60)
Do you sometimes have dyspnea (yes)	35 (58)	12 (52)	16 (59)	7 (70)
Do you often cough (yes)	26 (43)	15 (65)	9 (33)	2 (20)
Do you often have expectoration (yes)	22 (37)	7 (30)	11 (41)	4 (40)
Wheezing when not having a cold (yes)	25 (42)	7 (30)	15 (56)	3 (30)
Breathless when wheezing was present (yes)	22 (37)	7 (30)	11 (41)	4 (40)
Have you been woken up with a feeling of tightness in your chest (yes)	14 (23)	6 (26)	5 (19)	3 (30)
Have you been woken up by an attack of dyspnea (yes)	13 (22)	6 (26)	7 (26)	0 (0)
Have you been woken up by an attack of coughing (yes)	39 (65)	16 (70)	17 (63)	6 (60)
Do you have any nasal allergies (yes)	25 (42)	10 (43)	12 (44)	3 (30)
Do you often have common cold (yes)	22 (37)	8 (35)	8 (30)	6 (60)
Does it often take longer than 10 days to recover from a common cold (yes)	29 (48)	10 (43)	12 (44)	7 (70)
Do you smoke/Did you smoke (yes)	35 (58)	12 (52)	16 (59)	7 (70)
How much do you smoke [mean in pack year (SD)]	13 (15)	13 (10)	9 (10)	21 (26)

Spirometry was performed in the general practices by two research assistants, who were initially trained at the pulmonary outpatient clinic of the University Hospital of Heidelberg. In case of an unacceptable flow-volume curve, the spirometric software gave an error message. The best of three consecutive spirometry recordings was used, according to the guidelines of the American Thoracic Society (ATS) [14]. The maximal inspiratory and expiratory flow volume curves were generated by forced deep inspiration and expiration with short intervening periods of tidal breathing. The forced inspiratory manoeuvres were always done after forced expiratory manoeuvres.

Every patient received a bronchodilation test with an additional performance of spirometry 20 minutes after inhaling Salbutamol through a spacer. An obstructive airway disease was diagnosed if FEV_1_/VC ≤ 70%. The obstruction was classified as not reversible on Salbutamol, if the bronchodilation response Δ FEV_1 _was below 12% of the baseline and below 200 ml [15]. Blood was taken after the spirometry manoeuvre for determining CRP, which was assessed with an automated chemiluminometric assay kit.

Patients with no signs of airway obstruction after the results of the spirometry (i.e. FEV_1_/VC > 70%) received a PEF-meter (Vitalograph^®^) in order to perform PEF-measurements twice daily for 14 days. The patients were trained in performing this maneuver and documented the results in a diary. If the variability ((highest value - lowest value)/highest value) within these two weeks was above 20 %, reversible airway obstruction (= asthma) was diagnosed according to the international asthma guidelines [15]. The final diagnosis (reference standard) was confirmed by the main investigator (A.S.) and the pulmonologist (J.M.), who were kept unaware of the measured inspiratory parameters and CRP.

### Data analysis

As the sensitivities and specificities of clinical signs and symptoms and spirometric parameters are yet unknown for OAD diagnosed in primary care, a proper power calculation could not be performed. The data were analyzed with SPSS 11.0 for Windows.

The CRP values were log-transformed and entered the ROC-analyses to assess the best cut-off point. Sensitivity, specificity, and predictive values were calculated from 2 × 2 tables at the best cut-off-point. The best cut-off-point was chosen at the highest sum of positive and negative predictive value (PPV and NPV). CRP and clinical signs and symptoms were compared with the reference outcomes of spirometry and PEF-protocol. 95% confidence intervals were calculated using Wilson's method [16]. Binary logistic regression was done to estimate the diagnostic odds ratios (OR) of CRP and clinical signs and symptoms (as independent variables) for the presence of OAD (as dependent variable). Positive likelihood ratios (LR+) were calculated to receive the ratio of abnormal findings in ill and healthy patients. Negative likelihood ratios (LR-) were calculated for the ratio of normal findings in ill and healthy patients. 95% confidence intervals were derived from the log method [17].

In subgroup analyses, diagnostic values of CRP were calculated in combination with dyspnea, smoking history, coughing, expectoration, wheezing and abnormal finding in auscultation. In patients with the established diagnosis of OAD, the relationships between severity of dyspnea, CRP and spirometric parameters respectively were investigated using Spearman's correlation coefficient (for ordinal variables) and Pearson's correlation coefficient (for continuous variables).

## Results

### Study population

**Figure 1:**
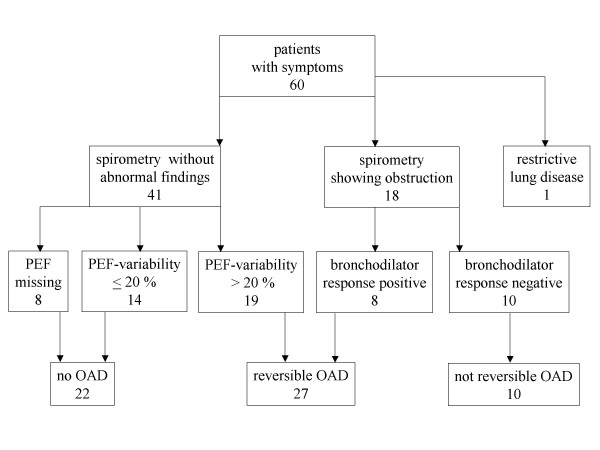
Flow chart of inclusion and diagnostic work up (OAD = obstructive airway disease; PEF = peak expiratory flow).

Two-thirds of the 60 patients were female; the average age was 46 years. 27 (45%) had a reversible OAD, of which 8 were identified by spirometry and 19 through the PEF-protocol (figure 1).

Ten showed an airway obstruction irreversible after inhalation of Salbutamol; one patient had a restrictive pattern. Fourteen (23%) patients with PEF-variability below 20 % within two weeks did not show abnormal signs in spirometry. Eight (13%) patients with inconspicuous spirometry did not bring back the PEF-protocol, assuring that they had no problems anymore. Thus, in total 37 (62%) did have OAD, whereas 23 (38%) patients were diagnosed as not having OAD.

The patients without OAD were four years younger than those with reversible OAD and the latter ones were eight years younger than those with not reversible OAD (table 1). The patients complained most frequently about wheezing (65%) and dyspnea (58%), coughing (43%) and expectoration (37%). Dyspnea and expectoration were mainly associated with OAD, whereas coughing was related with not having OAD. Wheezing was more pronounced for those having OAD. Waking up by an attack of coughing seems not to distinguish healthy from ill patients, as there is a similar distribution in all patients. Patients with non-reversible OAD seem to have a common cold (60%) more often, taking more than 10 days for recovering (70%). These patients also had the highest tobacco use (21 pack years). Those with reversible OAD had the lowest use (9 pack years).

### Diagnostic value of CRP and clinical signs and symptoms

The best cut-off point for CRP was at 2 mg/l, showing a diagnostic OR of 4.4 (table 2). The five symptoms with the best test characteristics are listed in table 2. Only self reported wheezing was significantly associated with having OAD (OR 3.4), showing a sensitivity of 75.7% and specificity of 52.2%. The sensitivity of "often coughing" was only 29.7% and the specificity was 34.8%. Thus, coughing was negatively related with OAD (OR 0.2). Moreover, auscultation was not significantly associated with the diagnosis.

**Table 2:** Sensitivities (sens), specificities (spec), positive and negative predictive values (PPV and NPV), odds ratios (OR) and likelihood ratios (LR+ and LR-) of clinical signs and symptoms and C-reactive protein (CRP) for the presence of obstructive airway disease (n = 60)

**Signs/Symptoms**	**Sens**	**95% CI**	**Spec**	**95% CI**	**PPV**	**95% CI**	**NPV**	**95% CI**	**OR**	**95% CI**	**LR +**	**95% CI**	**LR -**	**95% CI**
CRP > 2 mg/l	62.2	46.1–75.9	72.7	51.9–86.9	79.3	61.6–90.2	53.3	36.1–69.8	**4.4**	**1.4–13.8**	**2.3**	**1.1–4.7**	**0.5**	**0.3–0.8**
Dyspnea	62.2	46.1–75.9	47.8	29.2–67.0	65.7	49.2–79.2	44.0	26.7–62.9	1.5	0.5–4.3	1.2	0.8–1.9	0.8	0.4–1.4
Coughing	29.7	17.5–45.8	34.8	18.8–55.1	42.3	25.5–61.1	23.5	12.4–40.0	**0.2**	**0.1–0.7**	**0.5**	**0.3–0.8**	**2.0**	**1.1–3.7**
Expectoration	40.5	26.3–56.5	69.7	49.1–84.4	68.2	47.3–83.6	42.1	27.9–57.8	1.6	0.5–4.7	1.3	0.6–2.8	0.9	0.6–1.3
Wheezing	75.7	59.9–86.6	52.2	33.0–70.8	71.8	56.2–83.5	57.1	36.5–75.5	**3.4**	**1.1–10.3**	**1.6**	**1.0–2.5**	**0.5**	**0.2–0.9**
Smoking history	62.2	46.1–75.9	52.2	29.2–67.0	65.7	49.2–79.2	44.0	26.7–62.9	0.7	0.2–1.9	1.2	0.8–1.9	0.8	0.4–1.4
Auscultation	27.8	15.8–44.0	87.0	67.9–95.5	76.9	49.7–91.8	43.5	30.2–57.8	2.6	0.6–10.6	2.1	0.7–6.9	0.8	0.6–1.1

The diagnostic odds ratio of CRP increased when it was combined with the clinical symptom dyspnea (OR 8.5; 95% CI 1.7–42.3) or a positive smoking history (OR 8.4; 95% CI 1.5–48.9) (not in table). However, confidence intervals were wide because of small sample sizes (n = 35 in both subgroups). There was no significant improvement when CRP was combined with the other clinical signs and symptoms.

### Relation between severity of dyspnea, smoking history, CRP and spirometric parameters

There was a significant association between CRP and the severity of dyspnea in patients with established diagnosis (correlation coefficient_Spearman _[co_sp_] = 0.46) (table 3). There was also a significant relationship between severity of dyspnea and decrease of FEV_1 _(co_sp _= -0.52) and FIV_1 _(co_sp _= -0.41). CRP increased with the number of pack years (correlation coefficient_Pearson _= 0.648) (table 4).

**Table 3:** Relation between severity of dyspnea, CRP, FEV1 and FIV1 in patients with airway obstruction

**Severity**	**n**	**CRP [mg/l]** mean (sd)	**FEV**_1 _**[l]** mean (sd)	**FEV**_1_**pred. [%]** mean (sd)	**FIV**_1 _**[l]** mean (sd)	**Smoking [py]** mean (sd)
No dyspnea	14	2.8 (1.5)	3.4 (1.0)	94.6 (22.7)	3.65 (0.8)	4.4 (6.1)
Mild	16	7.1 (9.2)	2.4 (0.9)	83.8 (16.9)	2.9 (1.1)	9.9 (20.9)
Moderate	2	6.7 (1.1)	1.8 (0.4)	69.0 (21.8)	2.6 (0.1)	5.0 (7.1)
Severe	5	11.6 (13.1)	2.1 (1.1)	82.6 (16.0)	2.5 (1.2)	12.7 (8.2)
P		**0.004**	**0.001**	0.079	**0.012**	0.474

py = pack year

**Table 4:** Relation between smoking history, CRP, FEV1 and FIV1 in patients with airway obstruction

**Smoking [pack year]**	**n**	**CRP [mg/l]** mean (sd)	**FEV**_1 _**[l]** mean (sd)	**FEV**_1_**pred. [%]** mean (sd)	**FIV**_1 _**[l]** mean (sd)
0 < py ≤ 5	10	2.9 (1.6)	2.7 (1.0)	79.1 (22.0)	3.2 (0.1)
5 < py ≤ 10	3	3.2 (2.3)	3.0 (0.8)	89.8 (4.8)	3.5 (0.8)
10 < py ≤ 20	6	5.3 (4.2)	3.2 (1.4)	93.6 (14.6)	3.3 (1.1)
py > 20	4	13.7 (8.4)	2.7 (1.4)	82.1 (13.4)	3.3 (2.1)
p		**0.001**	0.381	0.552	0.147

## Discussion

This study demonstrates the difficulties in diagnosing obstructive airway diseases in general practice solely on the basis of clinical signs and symptoms. A combination of clinical signs and symptoms with CRP had the best diagnostic accuracy. CRP was related to smoking history; and it could be shown for the first time that CRP is related to the severity of dyspnea in patients with OAD. Also FEV_1 _and FIV_1 _were associated with the severity of dyspnea.

Only self reported wheezing was positively associated with the presence of the disease, but the other parameters of medical history were not accurate for identifying patients suffering from OAD. These findings are consistent with previous studies [18-20]. Interestingly, coughing is negatively associated with the presence of OAD. This might contradict common clinical findings [3], but these are often derived from selected clinical settings. Coughing also had a negative association in one survey, pointing out that this symptom is often attributed to other illnesses than asthma [18]. The results of auscultation are similar to those from Strauss et al, who found a positive LR of 2.7 in his survey. Thus, auscultation could be a difficult marker when the patient is not symptomatic during the physical examination.

The relation between CRP and the severity of airway obstruction in COPD was illustrated before in a population based survey, which found an association between FEV_1 _and CRP [8]. CRP has attracted more attention over the last years, as multiple associations with cardiovascular diseases [21], COPD [7], osteoporosis [22] and even depression [23] were identified. It is speculated that some of these diseases are caused by a low grade inflammation reflected by a small elevation of inflammatory markers [8], which might indicate a higher prevalence of systemic complications. Our findings also confirm that there is a chronic inflammatory process on a low level, since a no more than slightly elevated CRP (> 2 mg/l) has a significant diagnostic OR for OAD. This cut-off-point is in line with Sin et al. [8] who found their best threshold value for CRP at 2.2 mg/l. Our finding of a positive association between CRP and the number of pack years is supported by Gan et al., who detected that active smoking increases CRP [24]. They suggested that smoking and reduced FEV_1 _have an additive effect on systemic inflammation. The association with the severity of dyspnea and smoking history might make CRP of interest for risk stratification of patients with OAD in primary care. The importance of this has been underlined by Huijnen et al., who identified dyspnea as a significant predictor for mortality [25]. Until now, the diagnostic value of CRP for primary care was mostly evaluated for acute inflammatory diseases like lower respiratory tract infection (LRTI) [26] and acute maxillary sinusitis [27]. In LRTI, CRP has a high diagnostic value in combination with clinical signs and symptoms [26]. The impact on practice management was demonstrated as the use of CRP lowered antibiotic prescribing for sinusitis [28]. In our study the diagnostic odds ratios of CRP improved in patients with dyspnea or smoking history. However, the confidence intervals were wide, which could mainly be due to the small sample size. And it must be noted additionally that it was not possible to differentiate between asthma and COPD. Therefore, further studies in larger populations of patients with COPD and asthma are necessary.

We were the first to demonstrate in primary care that dyspnea is correlated with FIV_1_. This relation was so far only demonstrated in a highly selected population with already known COPD [11,12]. Taube et al found, that the perception of dyspnea is more related to the inspiratory than expiratory parameters [11]. Thus, FIV_1 _could also provide possibilities for risk stratification of patients in primary care as it is related with the severity of dyspnea. However, it must be noted for our study, that FEV_1 _shows a higher correlation coefficient than FIV_1_. Therefore the applicability and specificity of these parameters for primary care need to be evaluated in further studies.

There are some more limitations of our study. A reason for underestimation of the prevalence of OAD could be that eight patients did not bring back the PEF-meter with the protocol. However, they assured that they had no problems any more. One could argue that the performance of bronchoprovocation in a specialized centre would have been the best gold standard to evaluate the accuracy of spirometry in primary care and to distinguish between COPD and asthma. As this was not available in general practice, we tried to get as close as possible to the theoretical gold standard [29]. In a highly selected clinical population the measurement of PEF-variability within two weeks had a smaller sensitivity and specificity than bronchoprovocation [30]. However, the value of bronchoprovocation should not be over-estimated as a poor agreement between bronchial hyper-reactivity and clinical asthma was demonstrated in a review [31], and the correlation between the clinical diagnosis asthma and bronchoprovocation could be low [32]. Classical test characteristics derived from hospital studies are of limited value in primary care due to the lower incidence and smaller extent of the particular disease found there [33].

## Conclusion

The diagnostic accuracy of clinical signs and symptoms was low as only self-reported wheezing had a significant predictive value for the presence of OAD. The diagnostic accuracy of CRP improved in combination with dyspnea and smoking history. CRP was associated with the severity of dyspnea as well as FEV_1 _and FIV_1_. Furthermore, CRP increased with the number of pack years. Altogether, these parameters might allow risk stratification of patients with OAD in primary care. Further studies need to be done to confirm these findings.

## Competing interests

The author(s) declare that they have no competing interests.

## Authors' contributions

AS designed the study, performed the statistical analysis and wrote the manuscript. GJD helped with the analysis and to write the manuscript. IM performed the spirometry and helped with the data analysis. LG performed the spirometry and documented the data. FJM helped to interpret the data and to write the manuscript. JS supervised the study and contributed in the writing of the manuscript.

## Pre-publication history

The pre-publication history for this paper can be accessed here:


